# Processes and mechanisms of vegetation ecosystem responding to climate and ecological restoration in China

**DOI:** 10.3389/fpls.2022.1062691

**Published:** 2022-11-28

**Authors:** Tiantian Chen, Qiang Wang, Yuxi Wang, Li Peng

**Affiliations:** ^1^ Chongqing Key Laboratory of Surface Process and Environment Remote Sensing in the Three Gorges Reservoir Area, Chongqing Normal University, Chongqing, China; ^2^ Chongqing Field Observation and Research Station of Surface Ecological Process in the Three Gorges Reservoir Area, Chongqing Normal University, Chongqing, China; ^3^ Chongqing Institute of Surveying and Monitoring for Planning and Natural Resources, Chongqing, China; ^4^ College of Geography and Resources, Sichuan Normal University, Chengdu, China

**Keywords:** vegetation greening, nonlinear characteristics, climate, ecological restoration projects, normalized difference vegetation index (NDVI)

## Abstract

Vegetation is an essential component of the earth’s surface system and its dynamics is a clear indicator of global climate change. However, the vegetation trends of most studies were based on time-unvarying methods, cannot accurately detect the long-term nonlinear characteristics of vegetation changes. Here, the ensemble empirical mode decomposition and the Breaks for Additive Seasonal and Trend algorithm were applied to reconstruct the the normalized difference vegetation index (NDVI) data and diagnose spatiotemporal evolution and abrupt changes of long-term vegetation trends in China during 1982–2018. Residual analysis was used to separate the influence of climate and human activities on NDVI variations, and the effect of specific human drivers on vegetation growth was obtained. The results suggest that based on the time-varying analysis, high vegetation browning was masked by overall vegetation greening. Vegetation growth in China experienced an abrupt change in the 1990s and 2000s, accounting for 50% and 33.6% of the whole China respectively. Of the area before the breakpoint, 45.4% showed a trend of vegetation decrease, which was concentrated mainly in east China, while 43% of the area after the breakpoint also showed vegetation degradation, mainly in northwest China. Climate was an important driving force for vegetation change in China. It played a positive role in south China, but had a negative effect in northwest China. The impact of human activities on vegetation growthchanged from an initial negative influence to a positive one. In terms of human activities, an inverted-U-shaped relation was detected between CO_2_ emissions and vegetation growth; that is, the fertilization effect of CO_2_ had a certain threshold. Once that threshold was exceeded, it would hinder vegetation growth. Population density had a slight constraint on vegetation growth, and the implementation of ecological restoration projects (e.g., the Grain for Green Program) can promote vegetation growth to a certain extent.

## Introduction

Vegetation is a fundamental component of the terrestrial ecosystem that has played an important role in regulating regional climates, maintaining the surface energy balance, and so forth (Ballantyne et al., 2017; Forzieri et al., 2017; Pan et al., 2018). In the context of global environmental changes and intensified human activities, vegetation dynamics have been a research hotspot and received enhanced interest (Zhu et al., 2016; Zhao et al., 2020). The normalized difference vegetation index (NDVI), which measures the absorption and scattering of the vegetation canopy in the red and near-infrared bands (Hird and Mcdermid, 2009), has been a successful proxy for dynamic vegetation change (Piao et al., 2014; Liu et al., 2019; Wang et al., 2020). However, the time-series NDVI used by scholars often contains different frequencies, such as outliers, annual, and interannual trends (Verbesselt et al., 2010; Jong et al., 2011; Hawinkel et al., 2015) in addition to the noise caused by fog, clouds, and residual geometric errors (Roy et al., 2002; Yang et al., 2021). Also, recent time-series NDVI analyses often assumed that vegetation trends were monotonic over time, and the analysts have disregarded the nonlinearity and nonstationarity of NDVI trends (Piao et al., 2015). That might fail to accurately analyze the spatiotemporal variations in vegetation trends (Jong et al., 2012; Ji et al., 2014; Pan et al., 2018).

To overcome these problems, many temporal decomposition techniques have been recently proposed to detect short-term and long-term trends and residuals of time-series NDVIs. Examples of those techniques include the wavelet transform (Rhif et al., 2020), Fourier spectral analysis (Lhermitte et al., 2008), and detecting breakpoints and estimating segments in trends (DBEST) (Tomov, 2016). However, the wavelet transform is nonadaptive and cannot be integrated with other toolboxes (Hawinkel et al., 2015). Fourier spectral analysis and the DBEST application might require *a priori*-defined function to represent components and can recognize only short-term characteristics of vegetation NDVI changes. Consequently, the ensemble empirical mode decomposition (EEMD) has been proposed (Huang et al., 1998). It is an adaptive time-frequency analysis method suitable for decomposing time-series data and uses a quantitative method that focuses on interannual fluctuations (Hawinkel et al., 2015; Chen et al., 2017; Feng et al., 2021). To monitor nonlinear trends of vegetation change, piecewise linear regression (Pan et al., 2018), Theil–Sen estimation (Liu et al., 2015), the Mann–Kendall test (Martínez and Gilabert, 2009), polynomial fit (Jamali et al., 2014), and a Breaks for Additive Seasonal and Trend (BFAST) (Verbesselt et al., 2010) algorithm was developed. Unlike the other methods, the BFAST algorithm can monitor breakpoint positions in a time-series NDVI and determine the optimal number of breakpoints, has been robustly used with various data sources (Ma et al., 2020; Zhang et al., 2020).

Studies have found enhanced vegetation growth since the early 1980s in areas including northern tropical latitudes (Mao et al., 2016), karst areas (Tong et al., 2020; Zhang et al., 2021b), and part of Australia (Donohue et al., 2009). That growth might be the result of rising temperatures (Fensholt et al., 2012), the fertilization of CO_2_ (Piao et al., 2006; Zhu et al., 2016), and increased management intensity (Jia et al., 2014). However, some studies have also determined that reduced vegetation growth was hiding in overall vegetation growth (Fu et al., 2015; Feng et al., 2021), such as in northern Eurasia (Piao et al., 2011), the southwestern United States (Zhang et al., 2010), Amazonia (Doughty et al., 2015), and Inner Asia (Zhou et al., 2014). The reduced growth was caused by drought (Condorelli et al., 2018), harmful human activities, and so forth. On the whole, the leading causes of NDVI variation have been climatic forces such as temperature (TEM) and precipitation (PRE), and human activities such as socioeconomic development, land-use change, and implementation of ecological restoration projects (Wu et al., 2020a; Zhang et al., 2021b). How to quantify the relative effects of climate change and human activities on NDVI dynamic changes incurs several unique researches (Ge et al., 2021; Shi et al., 2020). Residual analysis, which is a quantitative method, can be used to identify the effects of climate and human activities on vegetation variation at spatiotemporal scales (Jiang et al., 2017; Chu et al., 2018; Zhang et al., 2021a), and has been used in this study.

**Figure 1:**
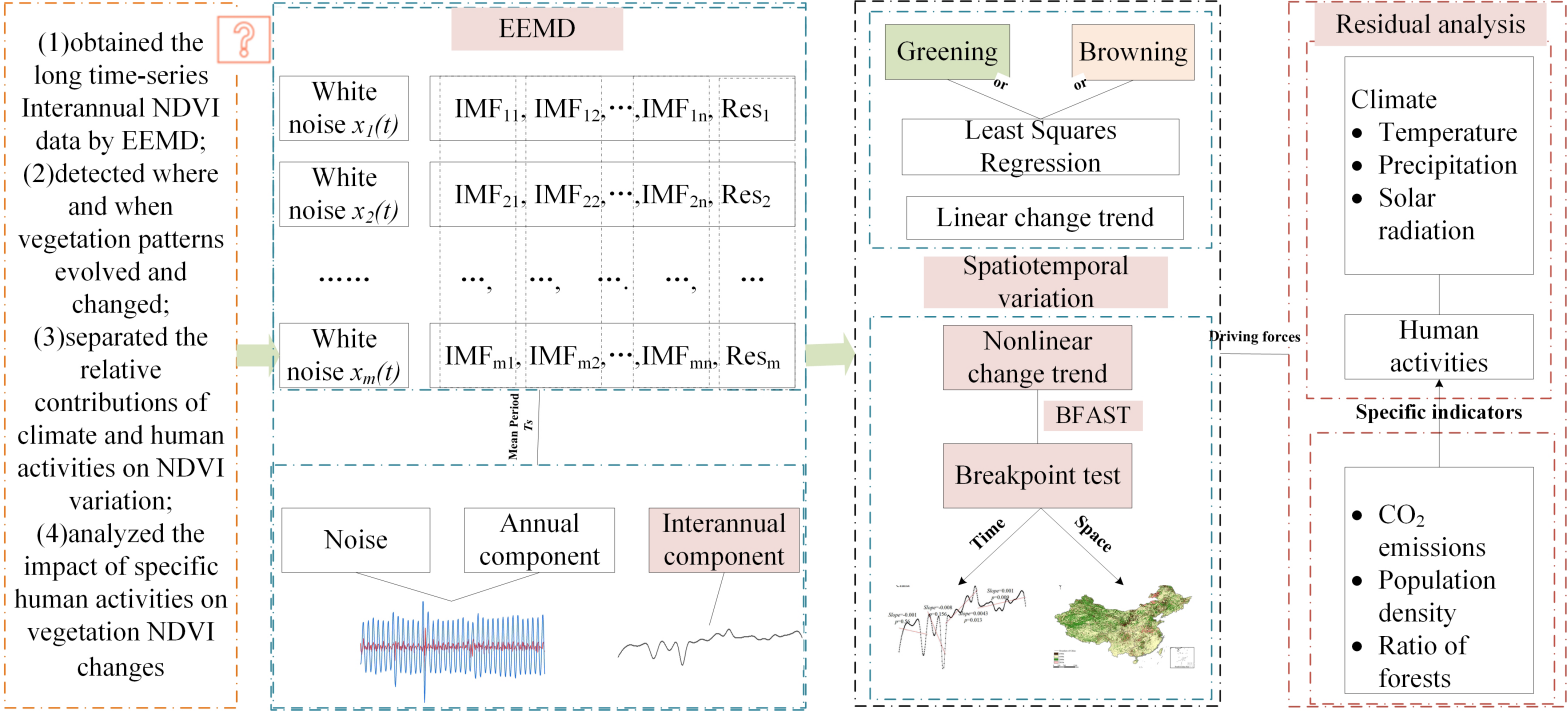
Framework map. *x(t)* means white noise, IMF means intrinsic mode functions, and Res is residual.

Many scholars have paid attention to the spatiotemporal change trends of the NDVI in China. Those studies found that since the 20th century, vegetation has improved significantly in the karst area of southwest China (Yue et al., 2020; Qi et al., 2021), in the Loess Plateau (Wu et al., 2020b; Li et al., 2021), and in northern China (Liu et al., 2021), largely due to ecological restoration projects. At the same time, however, drought has caused reduced vegetation growth in northwest China (Zhang et al., 2015). Most of those studies focused on short-term vegetation changes in specific regions, and less research has been done on long-term NDVI changes and their nonlinear characteristics over the whole of China. Studies have also paid much attention to the effect of climate on NDVI changes, but the effect of human activities especially the specific human drivers has rarely been quantified. For those reasons, in this study (1) long time-series NDVI data were obtained by EEMD (i.e., an interannual component), which represented actual vegetation growth; (2) it was detected where and when vegetation spatiotemporal patterns evolved and changed abruptly in China over nearly the last four decades using the BFAST algorithm; (3) residual analysis was used to evaluate and separate the relative contributions of climate and human activities on NDVI variation; and (4) three indicators were selected that indicated specific human activities, and the effect of specific human activities on vegetation NDVI changes was analyzed. This study can provide scientific support for a comprehensive understanding of the characteristics of vegetation change in China and an in-depth understanding of the effect of climate and human activities on vegetation change (**Figure 1**).

## Materials and methods

### Data sources

Currently, commonly used NDVI data products include the GIMMS3g NDVI, the SPOT-VGT NDVI, and the MODIS NDVI. The time series of the GIMMS3g NDVI data is longer (from July 1981 to December 2015) but lacks data for the recent 5 years, and its resolution is low (8 km). The SPOT-VGT and MODIS NDVI data have a high spatial resolution (approximately 1 km), but the time series is short, covering only approximately 20 years. The NDVI dataset used in this study was the NOAA CDR NDVI. Because the raw data for October, November, and December 1994 and November and December 2020 were missing for China, the data for those months were set at 0. Based on the NOAA CDR NDVI, the R rgee package was used to invoke the Google Earth Engine, then long-term time series for the NDVI in China were generated through a maximum-value composite procedure and clipping. The obtained NDVI data had the longest time series (from 1981 to 2020), and the spatial resolution was 5 km, which was better than that of the GIMMS3g NDVI data. The obtained data can be used for large-scale, long-term vegetation change research. The NDVI data in this study were from the National Earth System Science Data Center, National Science & Technology Infrastructure of China (http://www.geodata.cn).

In this study, TEM, PRE, and solar radiation (SR) were selected as the main climatic forces changing NDVI secular trends. The TEM and PRE datasets of China were generated from global 0.5° and high-resolution climate datasets through the Delta spatial downscaling scheme. Moreover, data from 496 independent meteorological observation points were used for verification, and the verification results were credible. They were from the National Tibetan Plateau Data Center (http://data.tpdc.ac.cn/zh-hans/), the time scale was from January 1901 to December 2020, and the spatial resolution was 1 km. The SR data in this study were derived from the dataset published by the Copernicus Climate Change Service (C3S), operated by the European Centre for Medium-Range Weather Forecasts. It can be downloaded at https://cds.climate.copernicus.eu/cdsapp#!/search. The SR data time scale was from January 1981 to February 2022, and the spatial resolution was 0.1°. Based on NDVI data, the coordinate system and resolution of the raster data were unified, and a raster dataset with a spatial resolution of 5 km from 1982 to 2020 was obtained.

The land-use dataset used in this study was to obtain the forests area and it was operated on the Google Earth Engine platform, with a time scale of 1990 to 2018 and a spatial resolution of 30 m. For details, please refer to Yang and Huang (2021). Long-term data on CO_2_ emissions and population density were obtained from the China Energy Statistical Yearbook and China Demographic Yearbook respectively. Other data, such as administrative boundaries, elevation, and the seven geographic regions of China (**Supplementary Figure S1**), were from the Resource and Environment Science and Data Center, Chinese Academy of Sciences (https://www.resdc.cn/).

### Methods

#### Extracting the interannual component from the time-series NDVI by EEMD

To avoid the mode aliasing problem of empirical mode decomposition, the EEMD was first proposed by Huang et al. (1998). It can decompose a time series (e.g., the NDVI, TEM, or sea level) into a series of physically meaningful components with no *a priori* functions while preserving the time domain flexibility of different time series (Hawinkel et al., 2015). The main goal of EEMD is to add white noise to the original data D(*t*) by using the statistical characteristics of Gaussian white noise with a uniform frequency distribution, so that the original data D(*t*) has continuity at different scales after adding the Gaussian white noise, thereby reducing the degree of modal aliasing and achieving signal decomposition. The specific decomposition steps of EEMD are (1) set the processing number of the original data D(*t*), (2) add random white noise to the original data and form a series of new data, (3) do empirical mode decomposition on the new data to obtain different intrinsic mode functions (IMFs) and a final residual term R(*t*) (Eq. [1]), and (4) average the IMFs of the corresponding modes. The EEMD decomposition result is thereby obtained. All IMFs must meet the following two conditions: (1) the number of extrema and zero crossings is less than 1 in the entire time series, and (2) the mean value of the upper and lower envelopes is 0 at any point. If those conditions are not met, the decomposition steps are repeated. After the EEMD, the low order is generally the high-frequency part, the high order is generally the low-frequency part, and each IMF not only contains different frequency components, but also carries different energy, and both vary with the original data D*(t)*.


(1) $$\text{D(}t\text{)=}\sum_{i=1}^{n}IMF_{i}(t)+\text{R}(t)$$

Meantime, after decomposing, all pixel values of the 37-year monthly NDVI data were spatially averaged, and a D(*t*) series with 444 values was obtained for China. Noises were iteratively added with 100 numbers and a 0.1 standard deviation to the D(*t*) series. Every IMF had a corresponding mean period *Ts*. According to Hawinkel et al. (2015), IMFs with a *Ts* of<0.5 year were considered noise components (*C_noise_*), IMFs with 0.5 year ≤ *Ts* ≤ 2.0 years were considered seasonal components (*C_annual_*), and IMFs with a *Ts* of >2.0 years and residual trends were considered interannual change components (*C_interannual_*).

#### Detecting breakpoints by the Breaks for Additive Seasonal and Trend algorithm

The BFAST algorithm can decompose an original time-series NDVI into seasonal components, trend components, and residual components and effectively detect sudden changes in a time-series NDVI (Verbesselt et al., 2012). In this study, the BFAST package was used in R to detect the number of breakpoints of an interannual NDVI and their positions in a long time-series NDVI. Specifically, first, a moving sum test was used to determine whether there was a mutation point, then the Bayesian information criterion was applied to determine the optimal number of breakpoints, and finally the locations of the breakpoints in the time-series NDVI were estimated through a least-squares regression.

In actual calculations, BFAST requires that the parameters of the seasonal model and the maximum number of breakpoints be specified. In terms of the parameters of the seasonal model, that model was removed when the interannual component of NDVI was obtained, so the seasonal model was set as “none.” In climatology, the interannual trend refers mainly to the variability of time scales greater than 1 year and less than 10 years. So, the maximum number of breakpoints was set to 3 in the time series. In terms of spatial distribution, to avoid the result being too complicated, the maximum number of breakpoints was set to 1.

#### Identifying the forces driving the interannual NDVI variation

Based on pixels, the absolute interannual change rate of NDVI was calculated by the unitary linear regression analysis method: the computation equation is expressed as


(2) $$\theta_{\text{slope}}=\frac{n\times \sum_{i=1}^{n}\left(i\times NDVI_{i}\right)-\sum_{i=1}^{n}i\sum_{i=1}^{n}NDVI_{i}}{n\times \sum_{i=1}^{n}i^{2}-{\left(\sum_{i=1}^{n}i\right)}^{2}}$$

where *n* is for times; *NDVI_i_* is the interannual NDVI of one pixel in time *i*; *θ*_slope >_0 indicates an increasing trend, and the converse denotes a decreasing trend; and |*θ*_slope_ *|* ≈ 0 shows that there is almost no change in the interannual NDVI.

In this study, a regression model between climatic forces (TEM, PRE, SR) and the interannual NDVI was constructed. The simulated NDVI according to the climate was called *NDVI_C_*. Then, the residual between the actual interannual NDVI and *NDVI_C_* was calculated and called *NDVI_H_* to characterize the effect of human activities on vegetation changes. The specific equation is


(3) $$NDVI_{C}=\beta_{0}+\beta_{1}\times PRE+\beta_{2}\times TEM+\beta_{3}\times SR+\epsilon$$

(4) $$NDVI_{H}=NDVI-NDVI_{C}$$

where *NDVI_C_* is the simulated NDVI under the effect of climate; *NDVI_H_* is the residual and represents the NDVI changes caused by human activities; *β_0_* is the regression intercept; *β_1_*, *β_2_*, and *β_3_* are the partial correlation coefficients between the NDVI and PRE, TEM, and SR; and ϵ is the random error. For the partial correlation coefficient, it represents the magnitude of the correlation between two variables calculated under the condition of eliminating the influence of other variables. Its equation is


(5) $$R_{xy}=\frac{\sum_{i=1}^{n}\left[(x_{i}-\bar{x})(y_{i}-\bar{y})\right]}{\sqrt{\sum_{i=1}^{n}{\left(x_{i}-\bar{x}\right)}^{2}}\sqrt{\sum_{i=1}^{n}{\left(y_{i}-\bar{y}\right)}^{2}}}$$

(6) $$R_{xy,z}=\frac{R_{xy}-R_{xz}R_{yz}}{\sqrt{\left(1-R_{xz}^{2}\right)}\sqrt{\left(1-R_{yz}^{2}\right)}}$$

where *R_xy_* is the linear correlation coefficient of the two variables of *x* and *y*, *R_xz_* is the linear correlation coefficient of the two variables of *x* and *z*, *R_yz_* is the linear correlation coefficient of the two variables of *y* and *z*, *R_xy,z_* is the partial correlation coefficient of *x* and *y* after the independent variable *z* is fixed, *x_i_* and *y_i_* are the values of the variables *x* and *y* in the time *i* respectively, $\bar{x}$ and $\bar{y}$ are the average value of the two variables *x* and *y* from 1982 to 2018, and *n* is the number of samples.

After achieving the separation of climate and human activities on vegetation growth, three indicators were selected to statistically clarify the effect of specific human drivers on vegetation changes: the amount of CO_2_ emission, population density, and the ratio of forests to China’s total area.

## Results

### Spatiotemporal variations in the interannual NDVI trend

**Figure 2:**
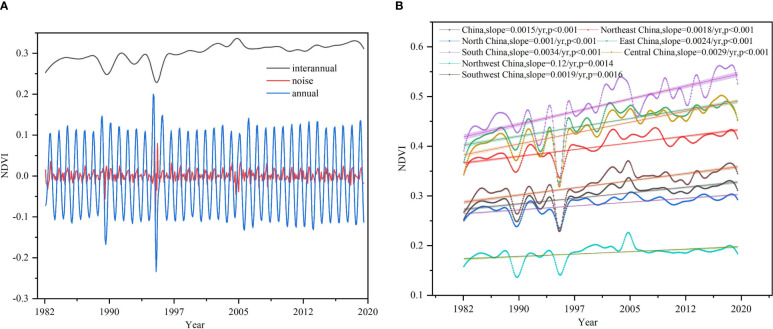
Decomposition result and change trend of interannual NDVI. **(A)** Change trend of noise, annual and interannual NDVI in China. **(B)** Change trend of interannual NDVI in China and its seven geographic regions.

The result of NDVI decomposition in this study was shown in **Figure 2A**. It can be seen that noise contained useless information, and the annual component represented the short-term seasonal trend. So, our focus was mainly on the interannual component’s spatiotemporal variations and driving forces. According to the statistics on the value of the interannual NDVI for all of China and its seven geographic regions, the interannual NDVIs for the seven geographic regions were significantly different. The NDVIs from large to small were south China > east China > central China > northeast China > southwest China > north China > northwest China (**Figure 2B**), and the maximum in south China was nearly 3 times the minimum in northwest China. There was also a noticeable interannual NDVI fluctuation and a significant increasing trend (*slope* = 0.0015 yr^–1^, *p<*0.001) from 1982 to 2018. The increasing rates of overall interannual NDVI trends of the seven geographic regions were different: they were significant at the 0.05 level for northwest China (*slope* = 0.12 yr^–1^) and southwest China (*slope =* 0.0019 yr^–1^), and were significant at the 0.001 level for south China (*slope* = 0.0034 yr^–1^), central China (*slope* = 0.0029 yr^−1^), east China (*slope* = 0.0024 yr^–1^), northeast China (*slope* = 0.0018 yr^–1^), and north China (*slope* = 0.001 yr^–1^).

**Figure 3:**
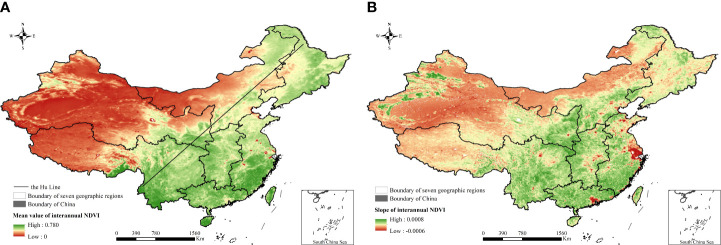
Mean value and change trend of multiyear interannual NDVI. **(A)** Mean value of multiyear interannual NDVI. **(B)** Change trend of multiyear interannual NDVI.

The mean value of interannual NDVI was between 0 and 0.78, it was spatially higher in southeast China than in the northwest, and the dividing line between high and low values was basically close to the Hu Line (**Figure 3A**). The slope of the interannual NDVI variation was between –0.0006 and 0.0008, and the slope of nearly 74.8% of the entire region was greater than 0 (**Figure 3B**), indicating vegetation greening in most parts of China, especially in central and south China. Nearly 25.2% of China showed a vegetation browning trend, which were mainly in northwest China.

### Breakpoints of NDVI trend

**Figure 4:**
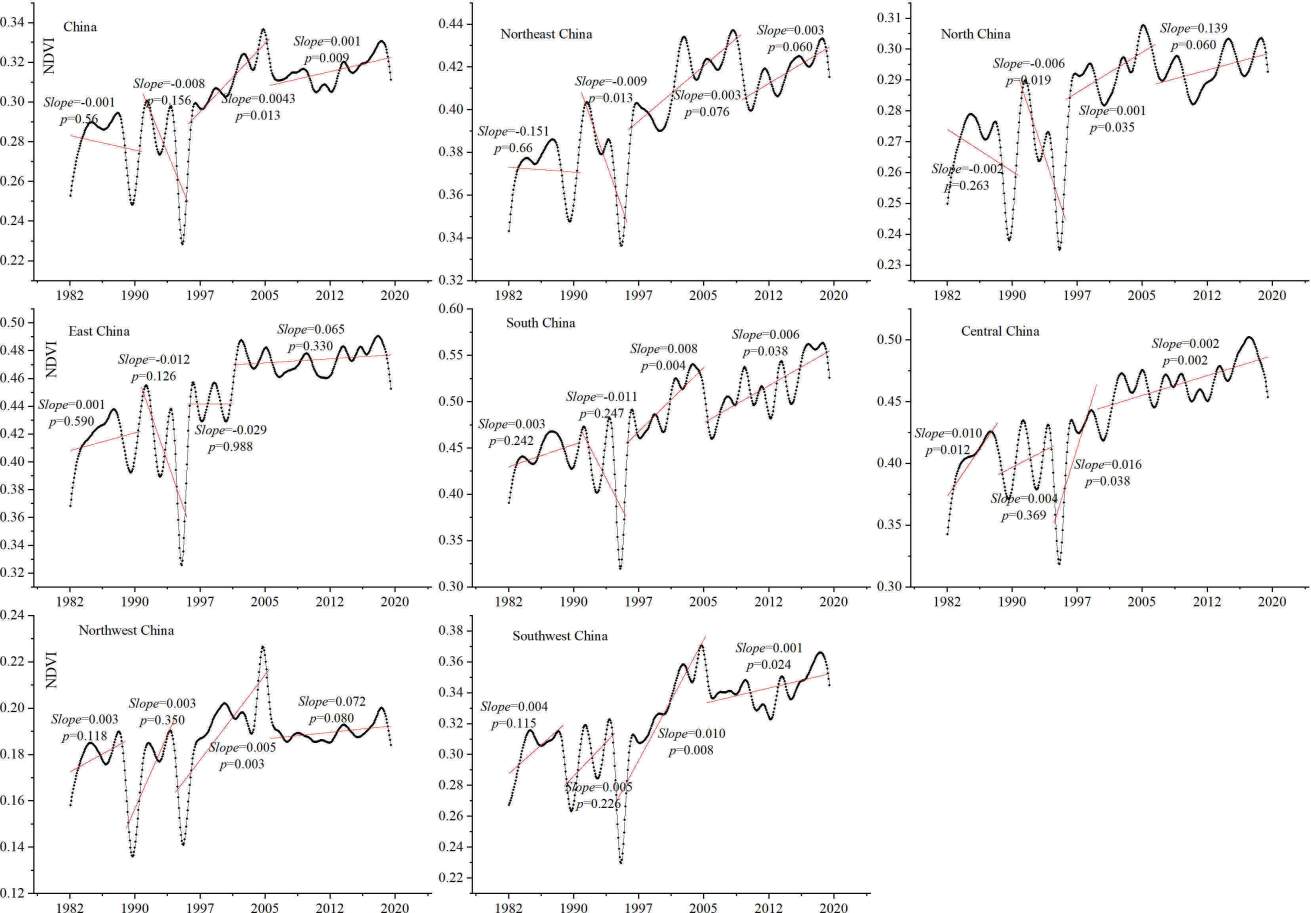
Breakpoints of time-series interannual NDVI in China.

The breakpoints of the time-series interannual NDVI were identified by the BFAST algorithm (**Supplementary Figure S2**), and the change trend of the interannual NDVI in four stages was linearly fitted (**Figure 4**). The results showed that the change of the interannual NDVI in China was clearly nonlinear. The interannual NDVI in the first and second stages showed a downward trend and in the later two stages showed an upward trend. An important turning point in the vegetation trend change in China was in 1995. Relative to the first stage (mainly from 1982 to 1990), the vegetation in the second stage (mainly from 1991 to 1995) had a larger downward trend (*slope* = 0.008, *p* = 0.156), and the growth rate of the vegetation (*slope* = 0.0043, *p* = 0.013) in the third stage (mainly from 1996 to 2004) was also greater than in the fourth stage (mainly from 2005 to 2018). For the seven geographical regions, the nonlinear characteristics of interannual NDVI changes were markedly different. In the first stage, the interannual NDVI in northeast and north China showed a decreasing trend, whereas east China, south China, central China, northwest China, and southwest China showed an increasing trend. In the second stage, northeast China, north China, east China, and south China all showed an NDVI decreasing trend, whereas central China, northwest China, and southwest China showed an increasing trend. In the third and fourth stages, the interannual NDVI basically showed an increasing trend in different geographic regions, but the rate was slightly different.

**Figure 5:**
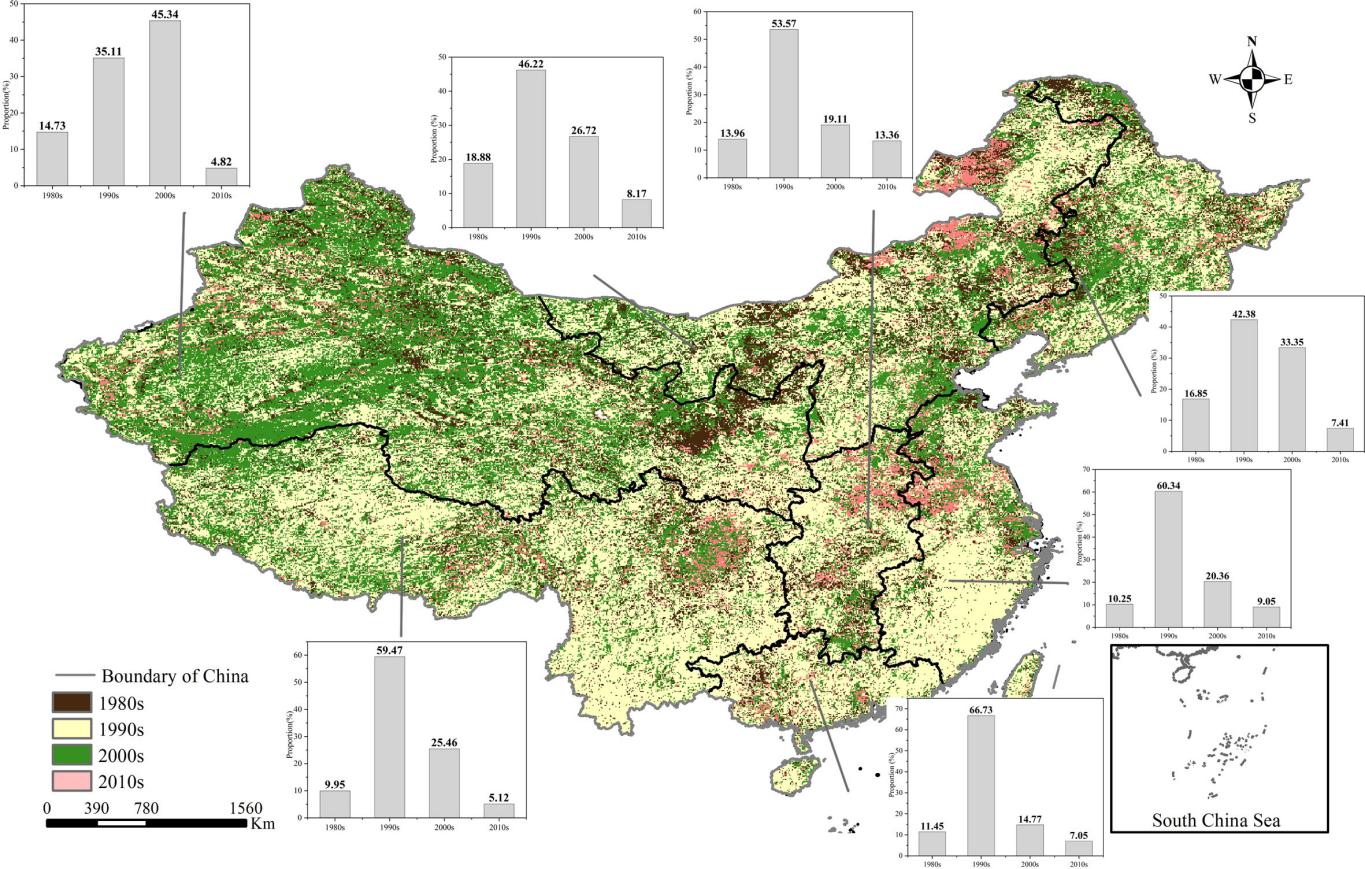
Spatial distribution of breakpoints of the interannual NDVI in China.

Half pixels of China showed breakpoints in interannual NDVI variations, most of which appeared in the 1990s, as shown in **Figure 5**, which were distributed in central and southern China. In fact, many relevant studies have found abrupt changes in NDVI trends during the 1990s (Chen et al., 2014), which was roughly in agreement with our studies. In the 2000s, 33.6% of China showed obvious breakpoints, mainly in northwest China. Also, the occurrence time of breakpoints in different geographical regions differed. In central China, east China, north China, south China, and southwest China, the abrupt change was mianly in the 1990s; its coverage in those regions accounted for 53.57%, 60.34%, 46.22%, 66.73%, and 59.47% of China respectively. For northeast and northwest China, the breakpoints of interannual NDVI changes were concentrated mainly in the 1990s and 2000s, accounting for 42.38% and 35.11%, and 33.35% and 45.34% of the total area respectively.

**Figure 6:**
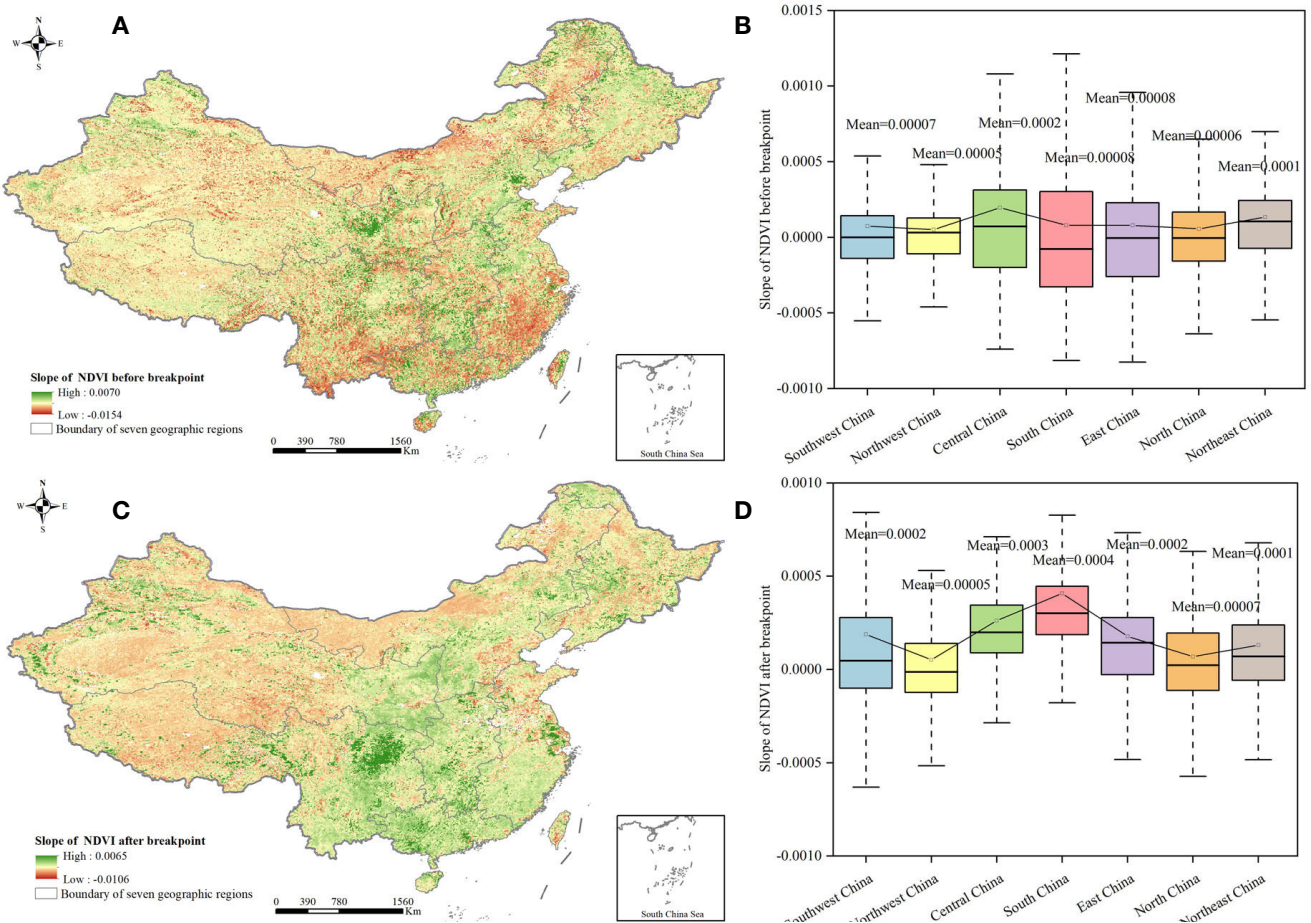
Change trends of interannual NDVI before and after breakpoint. **(A)** Slope of interannual NDVI before breakpoint. **(B)** Distribution of NDVI slope before breakpoint in different geographical regions. **(C)** Slope of interannual NDVI after breakpoint. **(D)** Distribution of NDVI slope after breakpoint in different geographical regions.

The slope of the interannual NDVI variation in 45.4% of China was less than 0 before the breakpoint, which meant that 45.4% of China had an NDVI degradation trend; those regions were distributed in the coastal area of southeast China (**Figure 6A**). And 54.6% of China had an NDVI recovery trend, those regions were distributed at the central part of China. For each geographic region, the slope of the interannual NDVI in south China before the breakpoint fluctuated greatly, whereas the mean value in central China was higher at 0.0002 (**Figure 6B**). After the breakpoint, the area where the slope of the interannual NDVI was less than 0 was slightly reduced to 43%, and the area where the slope was greater than 0 was in 57% of the total area, showing an increasing trend. The region with a slope of less than 0 was mainly in western China, and the region with a slope of greater than 0 was mainly in east China (**Figure 6C**). The slope of the interannual NDVI in southwest China after the breakpoint fluctuated greatly, whereas the mean value in south China was higher, at 0.0004 (**Figure 6D**). Overall, the change range of the interannual NDVI after the breakpoint was larger, and NDVI greening was stronger.

### Driving forces of interannual NDVI variation

**Figure 7:**
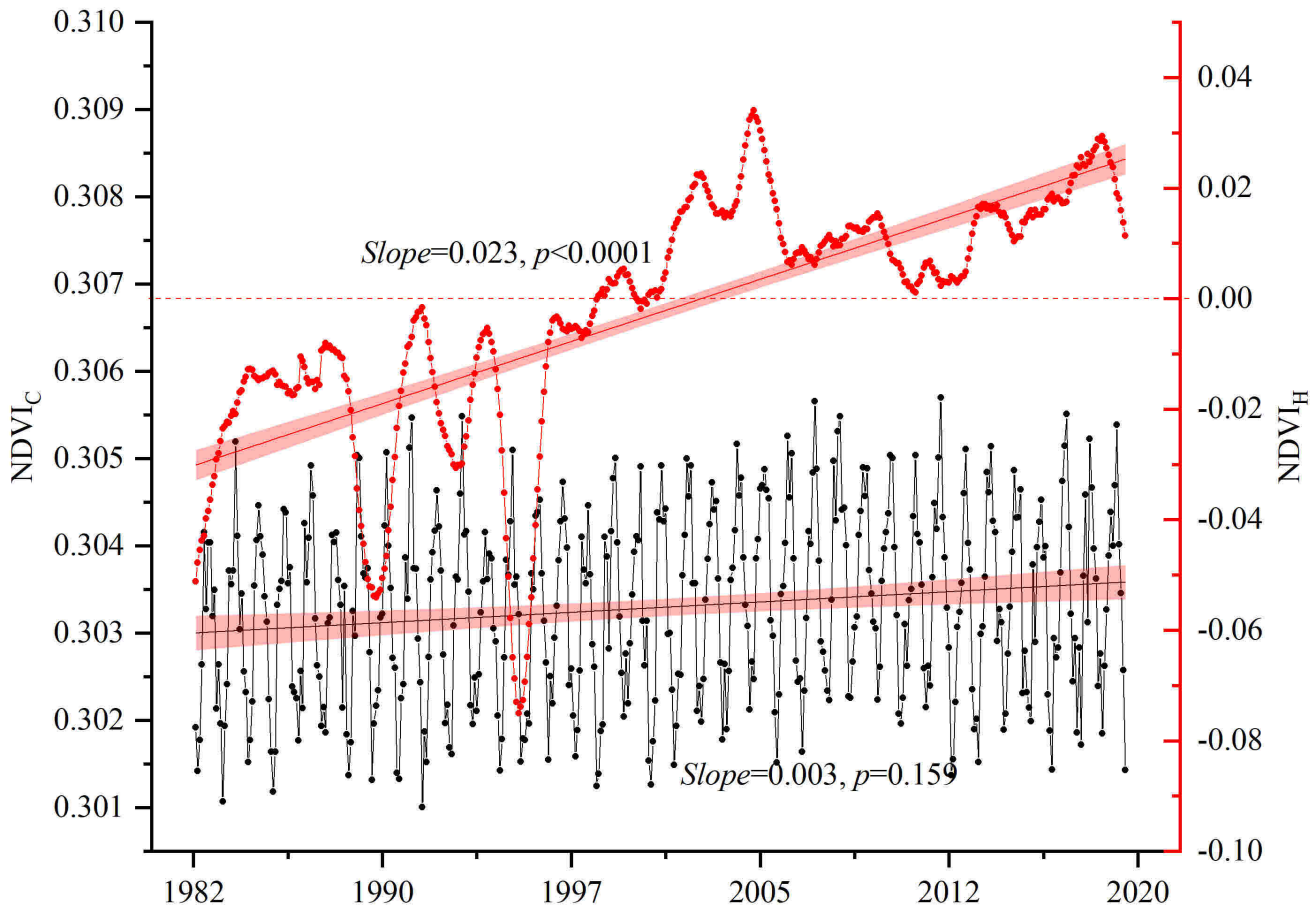
Long-term trend of *NDVI_C_* and *NDVI_H_*. *NDVI_C_* was the NDVI change caused by climate, and *NDVI_H_* was the NDVI change caused by human activities. The significance test is at the 0.05 level.

From the change trends of *NDVI_C_* and *NDVI_H_*, it can be seen that the mean value of *NDVI_C_* was greater than 0, indicating the positive effect of climate on vegetation growth in China (**Figure 7**). The effect of human activities on vegetation growth fluctuated greatly, and changed from a negative effect at the beginning to a positive effect at the end. At the same time, the mean value of *NDVI_C_* was substantially higher than that of *NDVI_H_*, indicating that climate had the greater effect on the variation in the vegetation NDVI in China. The turning point of *NDVI_H_* to a positive effect occurred in approximately 2008. In fact, China has done a series of ecological restoration projects since the 1990s, and the scale continued to expand in the 2000s. But the positive effect did not occur until 2008, which might be caused by other negative human drivers and reflect the lagging effect of human activities on vegetation growth.

**Figure 8:**
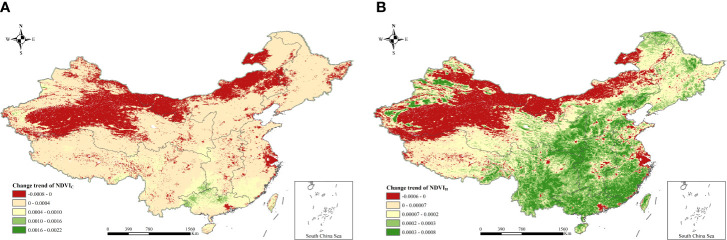
Slope of *NDVI_C_* and *NDVI_H_*. **(A)** Slope of *NDVI_C_*. **(B)** Slope of *NDVI_H_*.

The slope of *NDVI_C_* in north and northwest China was less than 0, indicating that the positive effect of climate on vegetation growth was weakening, and the restrictive effect was strengthening (**Figure 8A**). The slope of *NDVI_C_* in south China was the largest, indicating the continuous positive effect of climate on regional vegetation growth. As for the change of *NDVI_H_*, the areas with a slope of less than 0 were in north and northwest China, reflecting the sustainability of positive effects of ecological restoration projects on vegetation growth in those regions should be further strengthened (**Figure 8B**). Areas with a greater slope of *NDVI_H_* were mainly in central and south China, and many relevant studies have confirmed the contribution of Grain to Green program to vegetation greening in southern China (Tong et al., 2018), which was consistent with our study.

**Figure 9:**
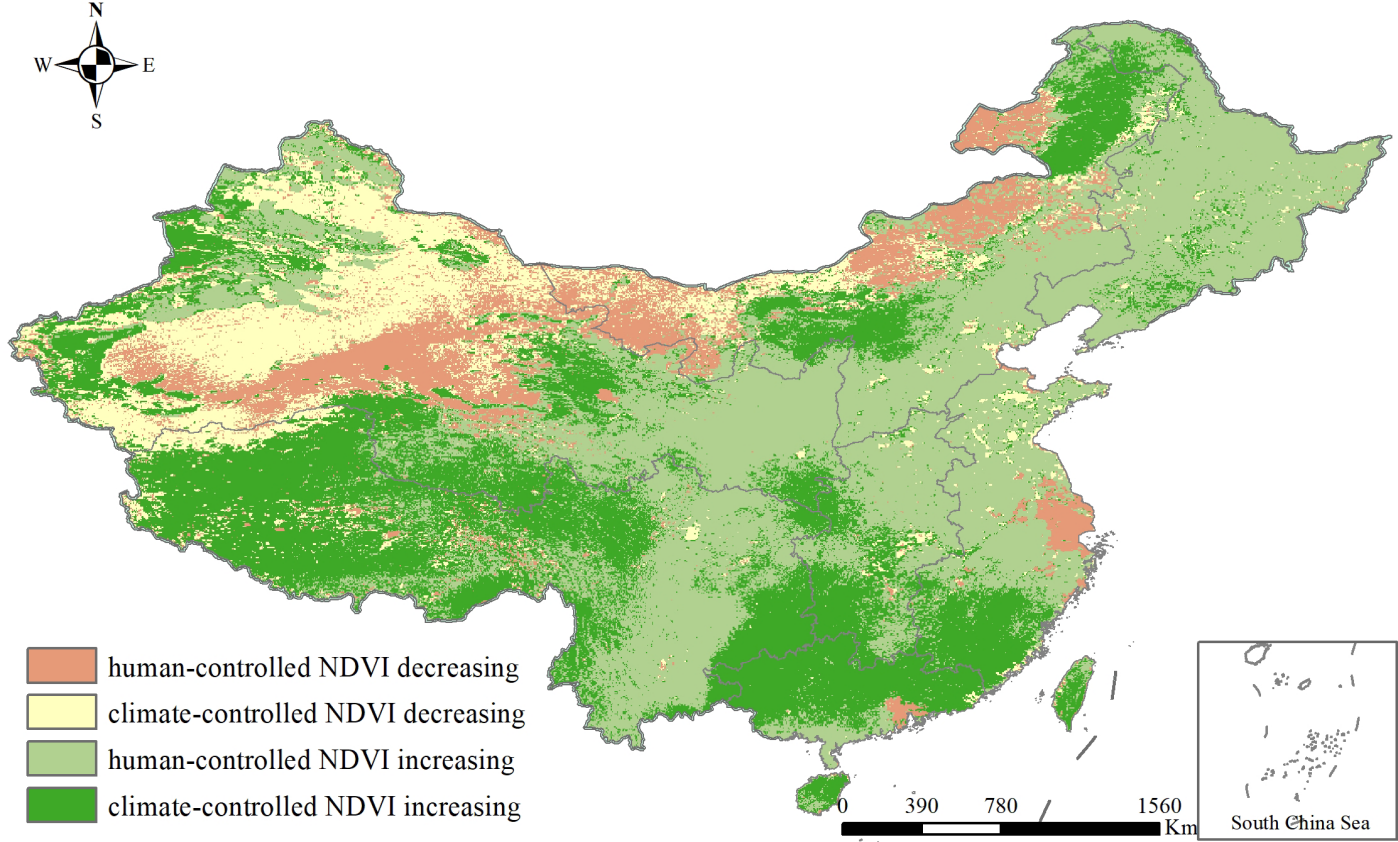
Driving-forces partition of interannual NDVI variation.

According to the driving-forces partition of NDVI variation shown in **Figure 9**, the areas of climate and human activities affecting NDVI changes were relatively close, indicating that the changes of interannual NDVI in China were the result of the combined effects of climate and human activities. Among them, the area of human-controlled decrease in the NDVI accounted for approximately 11.6% of the study area, distributed in mainly the northern margin of the Qinghai-Tibet Plateau, the northern part of north China, the Yangtze River Delta, and the Pearl River Delta. The area of climate-controlled decrease in the NDVI accounted for approximately 13.5% of the whole region, distributed mainly in northwest China. The area of human-controlled NDVI increase accounted for approximately 42.60% of the study area, located in mainly the central, northeast, and southwest parts of China, similar to the distribution of ecological construction projects. The area with a climate-controlled increase in the NDVI accounted for approximately 32.30% of the entire region, distributed in mainly the Qinghai-Tibet Plateau and south China.

For specific human drivers, the amount of CO_2_ emission represented economic development, population density represented the population agglomeration, and the ratio of forests represented the application of ecological restoration projects. It is shown in **Supplementary Figure S3** that there was a clear inverted-U-shaped relation between vegetation growth and CO_2_ emissions. That is to say, the increase in CO_2_ emissions at the initial stage stimulated vegetation growth. When the emissions peaked, the positive effect of CO_2_ emissions on vegetation growth reached the optimum, but when the emissions continued to increase, vegetation growth was inhibited. The threshold of CO_2_ emissions was roughly 4.6 to 4.8 million tons. Therefore, policies should be carried out to control the amount of CO_2_ emissions. Increased population is an important cause of land cover/use change and the consequent vegetation change. **Supplementary Figure S4** showed that there was indeed a negative correlation between population density and the interannual NDVI, but this negative effect was not substantial. As an important means to increase the vegetation coverage and solve the ecological environment problems in China, ecological restoration projects have always been a focus of research. From **Supplementary Figure S5**, it can be seen that the increase in the forested area ratio had a positive effect on vegetation growth in China. However, their relation gradually stabilized in the later period, which means that the increase in the ratio of forests area will no longer bring continuous vegetation growth, and the ecological restoration project needs to be optimized.

## Discussion

As an important proxy to characterize the earth vegetation coverage and analyze the response of vegetation to global climate change, the NDVI has been widely used in related fields (Julien and Sobrino, 2009; Saleem et al., 2020). However, the NDVI data used in many studies contained noise and seasonal components, and it was difficult to show the true change trends of vegetation. To this end, some methods have been proposed to decompose the NDVI data. Among them, the EEMD method is quite robust to noise and short-term disturbances and has been widely used (Pan et al., 2018). Based on this, this study used EEMD to reconstruct the NDVI and obtain its interannual components for a long-term trend study. In accordance with previous studies using time-unvarying methods, most vegetated lands have experienced overall greening over nearly four decades, and occurred in central and south China (Jong et al., 2011; Zhu et al., 2016). Actually, most of the browning trends were detected in this study by nonlinear methods, and this was supported by other studies at regional and continental scales (Cunha et al., 2015; Nyamekye et al., 2020; Valtonen et al., 2021). Some studies have pointed out that the 1990s was an important turning point for global vegetation changes (Wen et al., 2017; Zhang et al., 2021a), which is consistent with the results obtained in this study, where half pixels in China showed breakpoints in interannual NDVI variations in the 1990s. In terms of spatial distribution, the breakpoints appeared at different times in seven geographic regions. The mutation changes occurred in the 1990s in central, south, and east China, reflecting the distribution and development trend of China’s economy. However, the breakpoint of northwest China occurred mainly in the 2000s, which was in line with the vigorous implementation of ecological restoration projects in this region.

**Figure 10:**
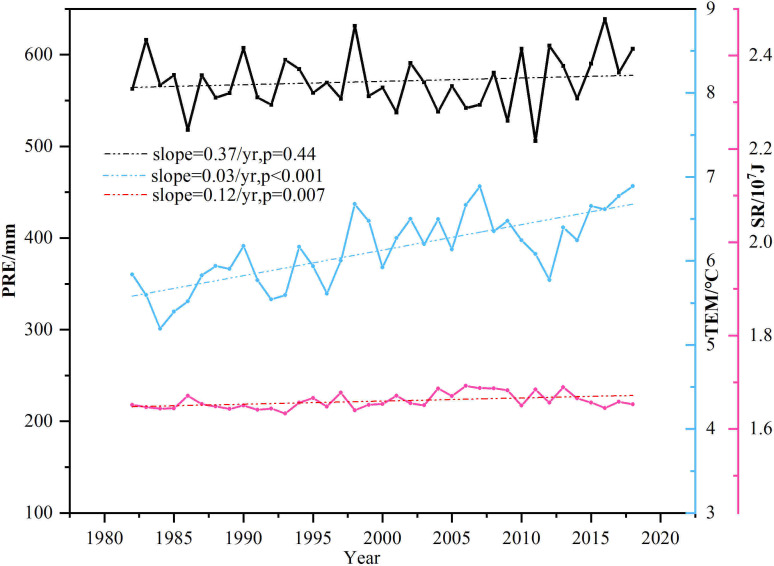
Temporal variations of interannual trend of climate in China.

This study argued that TEM, PRE, and SR were among the primary climatic forces affecting the distribution and growth of vegetation in China (Piao et al., 2006; Kong et al., 2017), and the interannual trends of the three climatic drivers in the same time period were obtained (**Figure 10**). It was found that TEM increased significantly in the 37 years (0.03 °C·yr^-1^, *P<*0.001), and there was an increasing trend in PRE and SR (0.37 mm·yr^-1^ and 0.12 10^7^J·yr^-1^), but they did not pass the significance test (*P =* 0.44 and 0.007). That is to say, the changes in PRE and SR in China were not obvious, but the TEM rose substantially. Increases in TEMs above the optimum will likely decrease vegetation photosynthesis and increase evaporation (Su and Shangguan, 2018; Feng et al., 2021). Especially for arid or semiarid regions, although these regions have implemented ecological restoration projects, the lack of PRE and the rise in evaporation hindered vegetation growth, showing a downward trend (Claussen et al., 2013). That was consistent with our results of vegetation browning in northwest China. In fact, climate and human activities jointly affected vegetation changes in China, and their influencing scopes were similar. Especially for the central China and karst areas of southwest China, human activities were the main driving forces for their vegetation increase, which is close to the related research and might be the result of the Grain for Green program (Tong et al., 2017; Liao et al., 2018; Tong et al., 2018). However, in the early stage of the implementation of the ecological restoration project, the growth rate of regional vegetation NDVI was relatively high (*slope* = 0.01, *P* = 0.008), but the growth rate decreased significantly in the later stage (*slope* = 0.001, *P* = 0.024), which means the sustainability of the ecological restoration program should be further strengthened. Also, the year that the effect of human activities on vegetation growth changed from negative to positive was 2008, which was late to the implementation time of the Grain for Green program. That is mainly because other human drivers may offset the positive effect of ecological restoration program on vegetation increase, and there is a lagged effecct of ecological restoration program on vegetation growth.

For specific human activities, the implementation of ecological restoration programs, which is characterized by increasing the area ratio of forests, has played a very important and positive role in increasing vegetation coverage at the early stage, and close to other studies (Tong et al., 2017; Hu et al., 2018); but the increase in forest area ratio did not bring a continuous enhanced vegetation growth. When the ratio of forests area reached a certain threshold, the vegetation NDVI tended to stabilize. Therefore, it is essential to reverse the single ecological restoration scheme, which regarded the forests area increasing as the main method and optimize the ecosystem structure. Also, as China’s industrialization process has accelerated, CO_2_ emissions caused by energy consumption have surged. Many studies have pointed out that as a raw material for vegetation photosynthesis, increasing CO_2_ emissions accelerates vegetation photosynthesis, and vegetation growth is promoted through fertilization (Wang et al., 2011). This study also found that CO_2_ emissions would promote vegetation growth through fertilization in the early stage, but this fertilization effect was not always continuous and effective. There was an inverted-U-shaped relation between the amount of CO_2_ emissions and the vegetation NDVI. That is, when the CO_2_ content exceeded a certain level, vegetation growth was inhibited, which partly reflected that ecosystems are suffering increasing stresses and experiencing a shift from a period dominated by the positive effects of fertilization to a period characterized by the saturation of the positive effects of fertilization on vegetation growth and rise of negative impacts of climate change (Peñuelas et al., 2017; Pan et al., 2018). That was mainly because CO_2_ content exceeding the threshold accelerates the rise of the surface TEM, prolongs the vegetattion growing season, and then aggravates the decomposition of organic matter (Terrer et al., 2019; Feng et al., 2021).

In this study, the breakpoints of vegetation NDVI change were identified by the BFAST algorithm. Actually, the identification of the breakpoint was related to the research periods and methods, and the difference in breakpoints number would cause different results, so comparing the conclusions of different research results must be more thorough. The function between interannual NDVI and TEM, PRE, and SR was constructed, and the NDVI changes caused by human activities were calculated by residual analysis. In fact, the NDVI changes resulted from the combined actions of climate and human activities, there was an obvious interaction between them, and the influence of the interaction often exceeded that of a single indicator (Wang et al., 2016). Therefore, research on the interaction between climate and human activities should be increased in the future.

## Conclusion

Through related methods, our study investigated the nonlinear characteristics of the interannual NDVI and its driving forces in China from 1982 to 2019. It was found by the time-unvarying method that there was a marked vegetation increase trend, with an average rate of increase of 0.0015 per year. The spatial distribution of vegetation NDVI in China was regular, and its high and low NDVI value boundary was close to the Hu Line but did not cross that line, which reflected the constraints of geographic features on vegetation growth. Actually, the vegetation growth in China was not monotonous, and substantial vegetation browning was masked by overall vegetation greening. For most regions, abrupt change occurred in the 1990s and 2000s: 45.4% of the area before the breakpoint showed a trend of vegetation decrease, whereas 43% of the area after the breakpoint also showed a vegetation decrease. Climate and human activities were the main forces for China’s vegetation changes. Specifically, human activities dominated the interannual vegetation recovery in central and south China, whereas climate was the leading cause of vegetation degradation in northwest China. In specific human activities, there was an inverted-U-shaped relation among CO_2_ emissions and vegetation NDVI changes, a negative effect of population density on vegetation increase, and a positive effect of the forest ratio on vegetation growth. Therefore, the results of this study provide a scientific basis for the development of vegetation management and protection strategies in the study area.

## Data availability statement

The original contributions presented in the study are included in the article/**Supplementary Material**, further inquiries can be directed to the corresponding author/s.

## Author contributions

TC: Conceptualization, Data curation, Writing – original draft. QW: Conceptualization, Data curation, Supervision, Writing– review and editing. YW: Data curation, Supervision, Writing – review and editing. LP: Conceptualization, Data curation, Supervision, Formal analysis, Writing – review and editing. All authors contributed to the article and approved the submitted version.

## Funding

This work was supported by the National Natural Science Foundation of China (No. 42001090) and the National Key Research and Development Program of China (2022YFF1300701).

## Acknowledgments

Acknowledgement for the data support from “National Earth System Science Data Center, National Science & Technology Infrastructure of China (http://www.geodata.cn)”.

## Conflict of interest

The authors declare that the research was conducted in the absence of any commercial or financial relationships that could be construed as a potential conflict of interest.

## Publisher’s note

All claims expressed in this article are solely those of the authors and do not necessarily represent those of their affiliated organizations, or those of the publisher, the editors and the reviewers. Any product that may be evaluated in this article, or claim that may be made by its manufacturer, is not guaranteed or endorsed by the publisher.
