# Supplementary material for: Processes and mechanisms of vegetation ecosystem responding to climate and ecological restoration in China
Source: Front Plant Sci. 2022 Nov 28;13:1062691. doi: 10.3389/fpls.2022.1062691 (PMC9742609; doi:10.3389/fpls.2022.1062691)
Supplement: Supplementary file 1 [file DataSheet_1.docx]

**Appendix materials S**

**Nonlinear characteristics of vegetation ecosystem and its response to climate and ecological restoration in China**

Tiantian Chen^1,2^, Qiang Wang^3^, Yuxi Wang^1^, Li Peng^4,*^

*^1^ Chongqing Key Laboratory of Surface Process and Environment Remote Sensing in the Three Gorges Reservoir Area, Chongqing Normal University, Chongqing 401331, China, ^2^ Chongqing Field Observation and Research Station of Surface Ecological Process in the Three Gorges Reservoir Area, Chongqing 401331, China, ^3^ Chongqing Institute of Surveying and Monitoring for Planning and Natural Resources, Chongqing 401121, China, ^4^ College of Geography and Resources, Sichuan Normal University, Chengdu 610101, China*

***** Corresponding author (Li Peng): pengli@imde.ac.cn


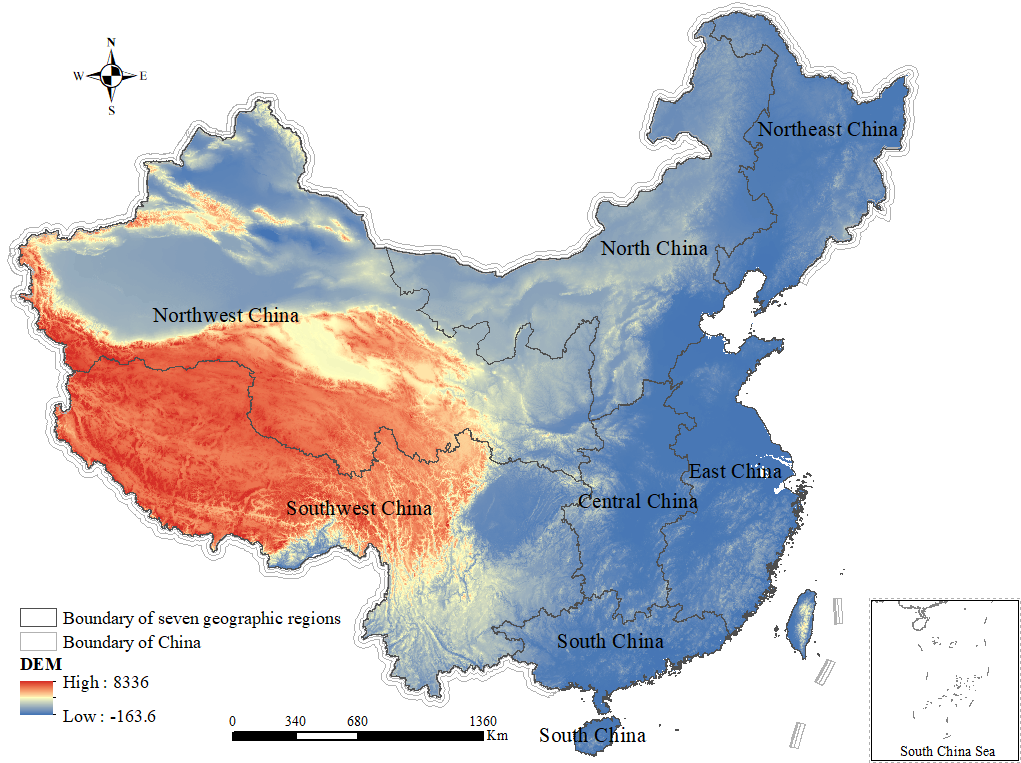


**FIGURE S1** Location map of study area

| 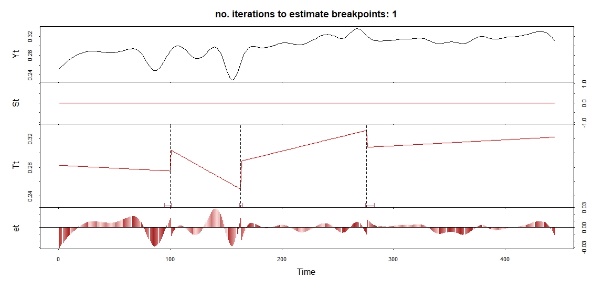 | 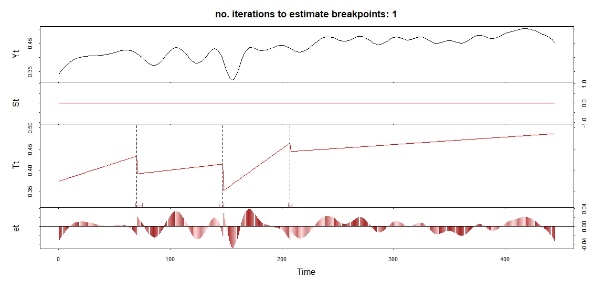 |
| --- | --- |
| China | Central China |
| 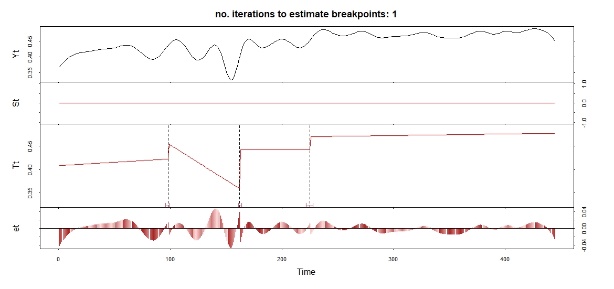 | 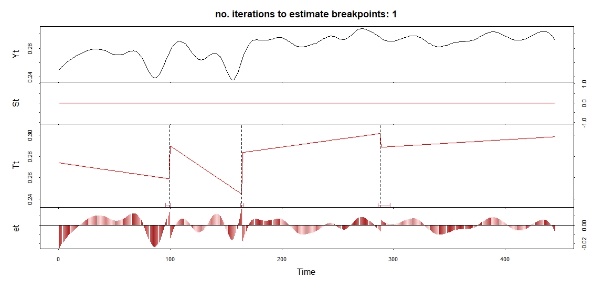 |
| East China | North China |
| 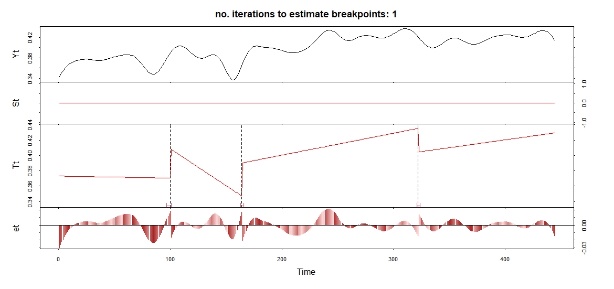 | 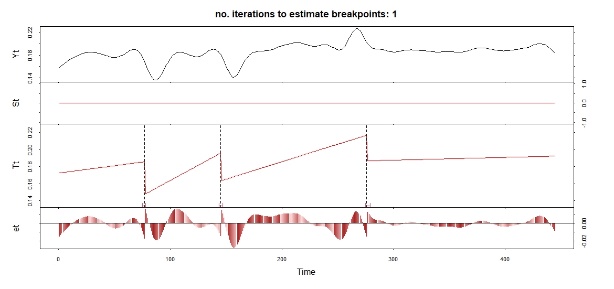 |
| Northeast China | Northwest China |
| 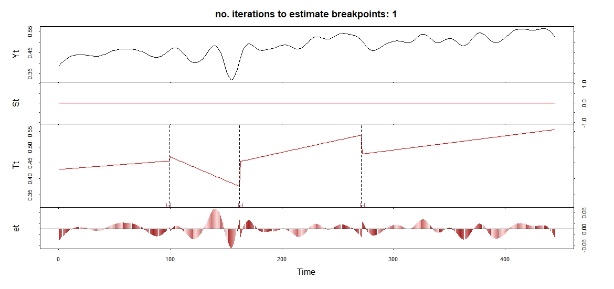 | 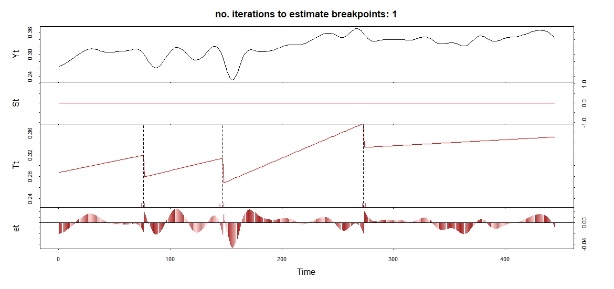 |
| South China | Southwest China |

**FIGURE S2** Breakpoint detection using the BFAST algorithm


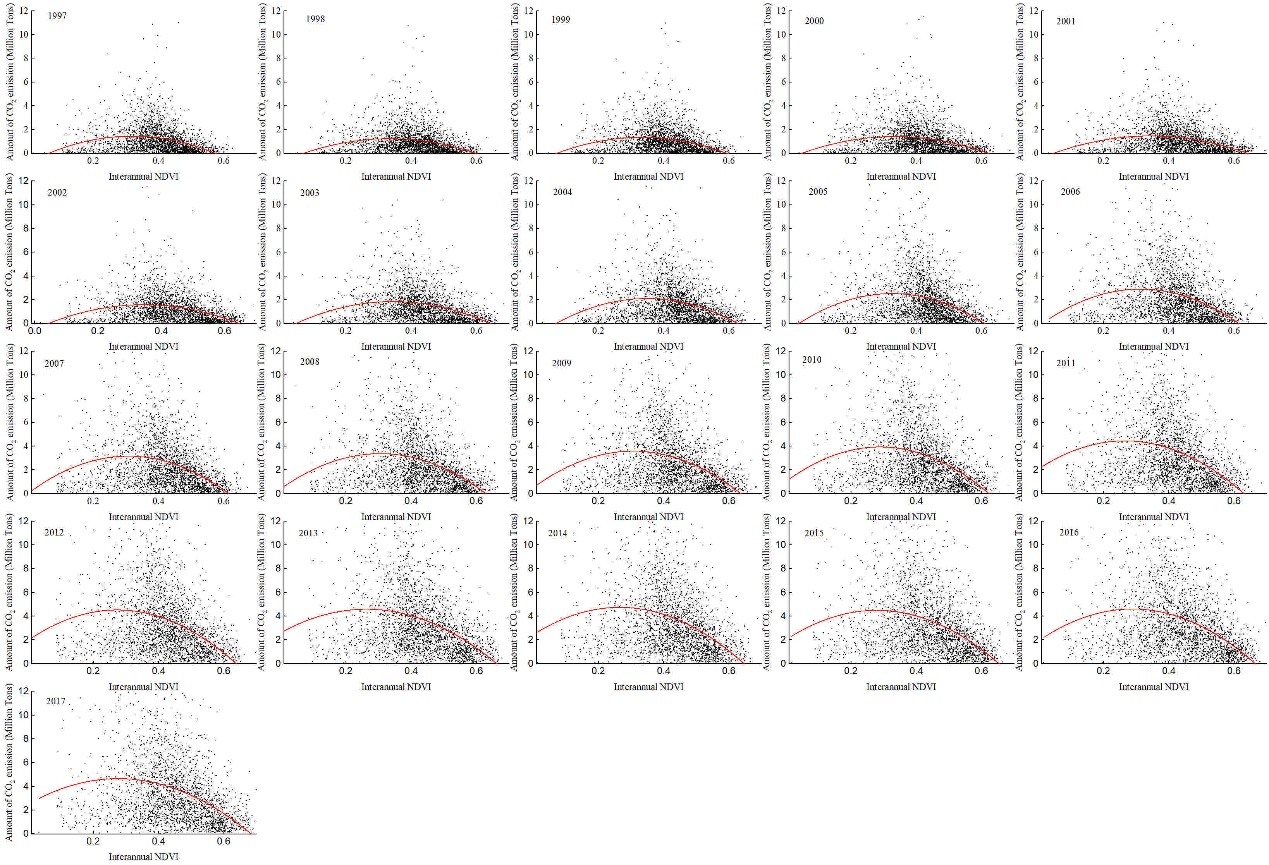


**FIGURE S3** Relationship between the amount of CO_2_ emission and NDVI


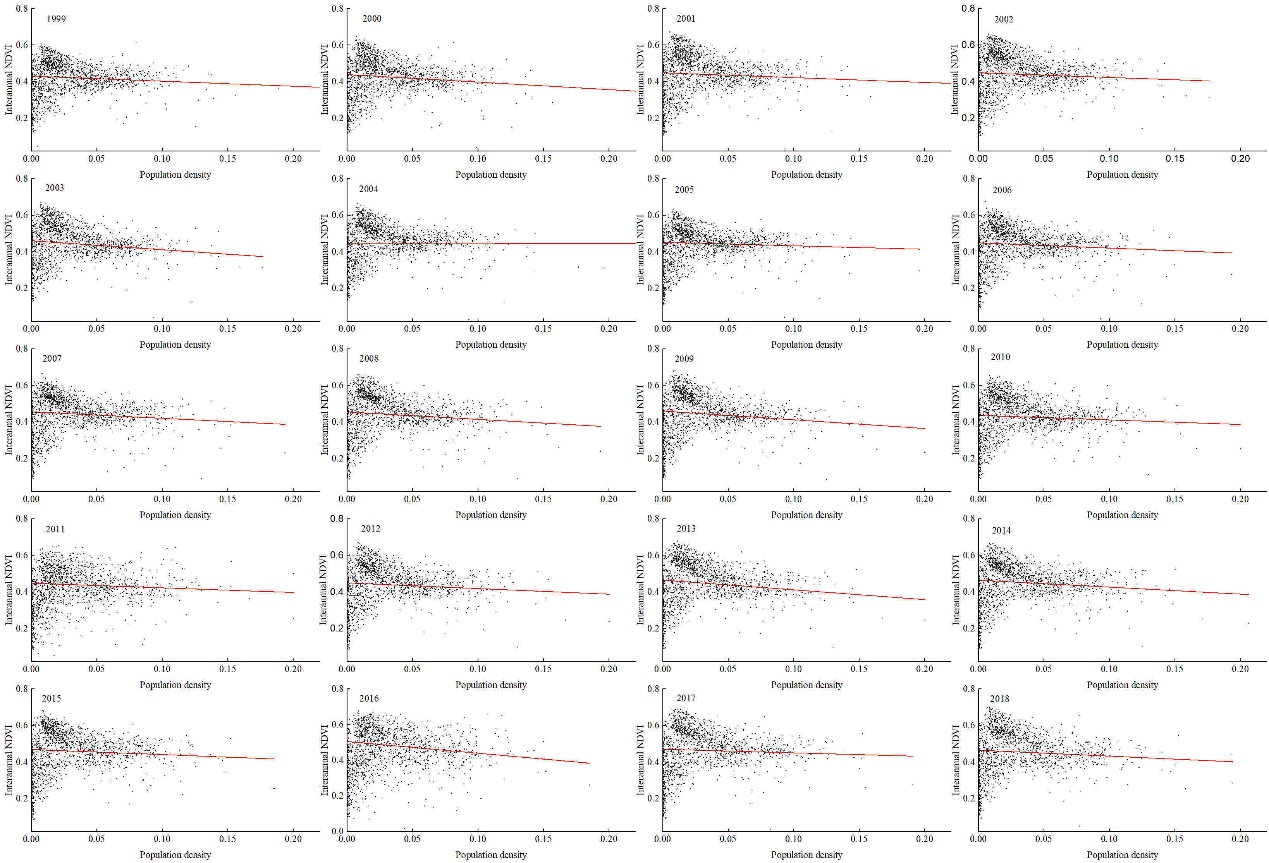


**FIGURE S4** Relationship between population density and NDVI


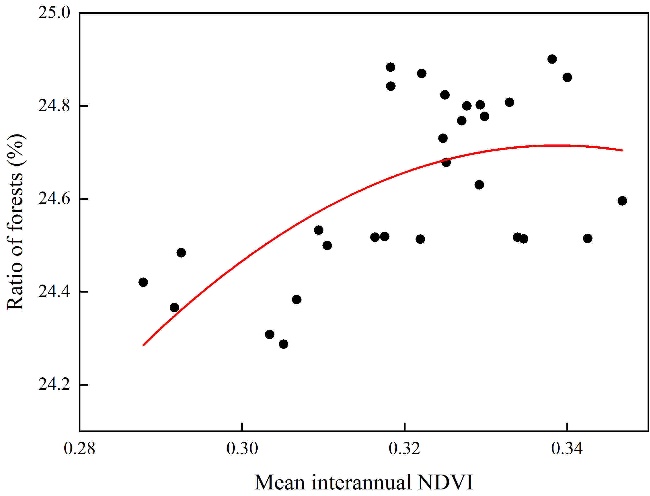


**FIGURE S5** Relationship between the ratio of forests and NDVI
